# Threats of Zika virus transmission for Asia and its Hindu-Kush Himalayan region

**DOI:** 10.1186/s40249-018-0426-3

**Published:** 2018-05-15

**Authors:** Meghnath Dhimal, Sushma Dahal, Mandira Lamichhane Dhimal, Shiva Raj Mishra, Khem B. Karki, Krishna Kumar Aryal, Ubydul Haque, Md Iqbal Kabir, Pradeep Guin, Azeem Mehmood Butt, Harapan Harapan, Qi-Yong Liu, Cordia Chu, Doreen Montag, David Alexander Groneberg, Basu Dev Pandey, Ulrich Kuch, Ruth Müller

**Affiliations:** 1 Nepal Health Research Council (NHRC), Ramshah Path, Kathmandu, Nepal; 2 Institute of Occupational Medicine, Social Medicine and Environmental Medicine, Goethe University, Frankfurt am Main, Germany; 3 Faculty of Social Sciences, Goethe University, Frankfurt am Main, Germany; 4 The University of Queensland, Brisbane, Australia; 5 Department of Public Health, Baldwin Wallace University, Berea, Ohio USA; 6 Department of Epidemiology, National Institute of Preventive and Social Medicine, Ministry of Health and Family Welfare, Dhaka, Bangladesh; 7 Public Health Foundation of India, Gurgaon, Haryana India; 8 Centre for Environmental Health, Gurgaon, Haryana India; 9 Translational Genomics Laboratory, Department of Biosciences, COMSATS Institute of Information Technology (CIIT), Islamabad, 45550 Pakistan; 10 Medical Research Unit, School of Medicine, Syiah Kuala University, Banda Aceh, Indonesia; 11 WHO Collaborating Centre for Vector Surveillance and Management, SKLID, CCID, ICDC, China CDC, Beijing, China; 12 Centre for Environment and Population Health, Griffith University, Nathan, Queensland Australia; 13 Barts and the London School of Medicine, Centre for Primary Care and Public Health, Queen Mary University of London, London, UK; 14 Department of Health Services, Ministry of Health, Government of Nepal, Kathmandu, Nepal

**Keywords:** *Aedes aegypti*, *Aedes albopictus*, Dengue virus, Chikungunya virus, Hindu Kush Himalayas, Mountain, Poverty, Zika virus

## Abstract

Asia and its Hindu Kush Himalayan (HKH) region is particularly vulnerable to environmental change, especially climate and land use changes further influenced by rapid population growth, high level of poverty and unsustainable development. Asia has been a hotspot of dengue fever and chikungunya mainly due to its dense human population, unplanned urbanization and poverty. In an urban cycle, dengue virus (DENV) and chikungunya virus (CHIKV) are transmitted by *Aedes aegypti* and *Ae. albopictus* mosquitoes which are also competent vectors of Zika virus (ZIKV). Over the last decade, DENV and CHIKV transmissions by *Ae. aegypti* have extended to the Himalayan countries of Bhutan and Nepal and ZIKV could follow in the footsteps of these viruses in the HKH region. The already established distribution of human-biting *Aedes* mosquito vectors and a naïve population with lack of immunity against ZIKV places the HKH region at a higher risk of ZIKV. Some of the countries in the HKH region have already reported ZIKV cases. We have documented an increasing threat of ZIKV in Asia and its HKH region because of the high abundance and wide distribution of human-biting mosquito vectors, climate change, poverty, report of indigenous cases in the region, increasing numbers of imported cases and a naïve population with lack of immunity against ZIKV. An outbreak anywhere is potentially a threat everywhere. Therefore, in order to ensure international health security, all efforts to prevent, detect, and respond to ZIKV ought to be intensified now in Asia and its HKH region. To prepare for possible ZIKV outbreaks, Asia and the HKH region can also learn from the success stories and strategies adopted by other regions and countries in preventing ZIKV and associated complications. The future control strategies for DENV, CHIKV and ZIKV should be considered in tandem with the threat to human well-being that is posed by other emerging and re-emerging vector-borne and zoonotic diseases, and by the continuing urgent need to strengthen public primary healthcare systems in the region.

**Electronic supplementary material:**

The online version of this article (10.1186/s40249-018-0426-3) contains supplementary material, which is available to authorized users.

## Multilingual abstract

Please see Additional file 1 for translations of the abstract into the five official working languages of the United Nations.

## Background

Since the report of the first active circulation of Zika virus (ZIKV) in the western hemisphere in early 2015 in Brazil [1], the virus has been spreading rapidly through different transmission pathways, *Aedes* mosquito bite being the predominant path. Recently, 85 countries, territories or subnational areas have been reported to have evidence of mosquito borne ZIKV transmission [2]. Human-to-human transmission of ZIKV through sexual contact was reported in Colorado USA in 2008 when a scientist contracted the infection during his work in Senegal and transmitted it to his wife upon his return [3] and since February 2016, 13 countries have already reported ZIKV transmission possibly via sexual contacts [4]. The number of countries and territories reporting the cases of microcephaly and/or central nervous system malformation, and Guillain-Barré syndrome potentially associated with ZIKV has reached 31 and 23 respectively [4]. Supported by a fast-growing body of scientific evidence corroborating the significant public health threats of ZIKV, the World Health Organization (WHO) declared the ZIKV epidemic as an international public health emergency at the beginning of 2016 [5]. However, health systems and outbreak preparedness are responding only slowly in certain regions. In this respect, we are particularly concerned about Asia and its Hindu-Kush Himalayan (HKH) region because the HKH region is particularly vulnerable to environmental change, especially climate and land use changes and further influenced by rapid population growth, high level of poverty and unsustainable development [6–8]. The HKH region extends 3500 km including all or some parts of eight countries ranging from Afghanistan in the west to Myanmar in the east [9]. It comprises approximately 18% of the world’s mountain areas including all the area of Nepal and Bhutan, 60% of Afghanistan, 51% of Pakistan, 47% of Myanmar, 17% of China, 14% of India and 9% of Bangladesh [6, 9]. The rate of atmospheric warming in the Himalayas has been reported to have been much greater (0.06 °C yr.^− 1^) than the global average in the last three decades [10].

### Mosquito-borne viral diseases in Hindu-Kush Himalayan region

Despite its mountainous terrain, the HKH region is now endemic to at least four major mosquito-borne viral diseases (MBVDs): Japanese encephalitis, West Nile fever, chikungunya and dengue fever [11]. The already established distribution of human-biting mosquito vectors and a naïve population with a lack of immunity against ZIKV places HKH region at a higher risk of ZIKV. Some of the countries in the HKH region have already reported ZIKV cases, for example, Bangladesh confirmed its first case of ZIKV in March 2016 in a 67 years old person who had never left the country [12], and Myanmar detected its first ZIKV infection in a pregnant foreign woman in late October 2016 [13]. China reported an imported ZIKV case of a 28-year-old man who had been working in Central America for the last 2 months as the first ZIKV case in the Henan province [14]. India also reported three laboratory-confirmed cases of ZIKV in May 2017 [15]. These cases in India do not have any history of travel to ZIKV-affected countries suggesting possible local transmission [15, 16]. Moreover, confirmation of autochthonous spread of ZIKV in neighboring countries such as Singapore in August 2016 [17] and report of ZIKV-related microcephaly cases in Thailand [18] has also substantially increased the risk of locally acquired or imported ZIKV in the HKH region primarily because of multiple modes of travel (air, land, sea) between different countries in the region. For instance, a recent study suggests that air traffic has played a more important role than maritime transport in shaping large-scale geographic dispersal of dengue virus (DENV) in Asia and the patterns of DENV spread result from the interplay between intensity and structure of human mobility through the air transportation network [19]. Similarly, the increasing threat of dengue and epidemic resurgence to local population and global community has increased with rapid unplanned urbanization and the growing global trade and travel [20, 21]. As ZIKV infection has a similar epidemiology and transmission cycle as DENV and chikungunya virus (CHIKV), infections and dispersal routes may also be similar. International travel and trade could be another route of ZIKV entry in Asia. For instance, India is developing more trade relationships with ZIKV-affected countries where prostitution is legal, which can be an organized channel for the route of ZIKV entry in Asian region [22].

It is reported that global geographical expansion of ZIKV is enabled by climatic conditions suitable to support the population growth of *Ae. aegypti* and *Ae. albopictus* mosquitoes and increased globalization continues to pose a risk for the spread of infection [23]. The potential risk of local transmission of ZIKV and the threat of outbreaks is equal for all the countries where mosquitoes susceptible to or competent for ZIKV are found and are endemic for dengue, yellow fever and chikungunya [23]. A recent study shows that the probability of ZIKV outbreaks increases with vapor pressure, the occurrence of DENV, and population density in Asia-Pacific regions [24]. Phylogenetically, two distinct lineages of ZIKV exist: the African lineage and the Asian lineage [25]. The reported cases of ZIKV in HKH region are presented in Table 1. Similarly, the risk factors for ZIKV transmission in Asia and HKH region are summarized in Table 2.

**Table 1:** Reported cases of Zika virus in HKH region

HKH countries	Total number of ZIKV reported	Month/Year
Afghanistan	NA	
Bangladesh	1	March 2016
Bhutan	NA	
China	1	February 2016*
	1	September 2016
India	3	May 2017
Myanmar	1	October 2016
Nepal	NA	
Pakistan	NA	

*a man from Jiangxi province was infected with the virus during a trip to Venezuela in late January and was treated there in Venezuela before travelling home

**Table 2:** Risk factors for Zika virus transmission in Asia and Hindu Kush Himalayan region

Region	Risk factors	Sources
Asia	Intense urbanization, increasing high population density, tropical climate, poor waste management, cross reaction and diagnostic difficulties, underreporting, co-circulation of dengue and chikungunya virus, establishment of *Aedes* vectors, globalization of travel and trade, poverty, vector occurrence probability, per capita health expenditure, virus strain, urban-rural set-up, vapor pressure, GDP, number of travelers, altitude, temperature, land cover	[19–24, 28, 33]
Hindu Kush Himalayan region	Shifting of *Aedes* vectors in highlands, frequent movement of people to Zika virus endemic areas, climate change, land use change, naïve population with a lack of immunity against Zika virus, poverty, higher warming rate than elsewhere in the region	[6–8, 10, 27–34, 38]

### Climate change and vector adaptation in the Hindu-Kush Himalayan region

CHIKV and DENV are transmitted by *Ae. aegypti* and *Ae. albopictus* mosquitoes which are also competent vectors of ZIKV [26]. Theses vectors are broadly established up to at least 2000 m above sea level in the HKH region [27–30]. The broad co-occurrence of *Ae. aegypti* and *Ae. albopictus* in the HKH region increases the risk for MBVDs transmission [29, 30]. Climate change is an ongoing phenomenon and it does not look promising that its negative impacts will be minimized soon, if at all. These changes are linked to an increased transmission window for mosquito-borne diseases thereby increasing population vulnerability [31, 32].

It is anticipated that ZIKV will continue to spread rapidly where competent vectors are found and major epidemics of ZIKV infection may occur globally. Importantly, human activity and its consequences like the globalization of trade and travel, rapid unplanned urbanization, and climate change increase the availability of breeding sites for ZIKV vectors, their spread to new areas, and their likelihood of establishing stable populations in newly colonized environments [33]. According to a recent study, there is an increasing trend of the epidemic potential and length of the transmission season of MVBDs under different climate change scenarios leading to the occurrence of dengue in temperate regions and tropical highlands [34]. Moreover, the Intergovernmental Panel on Climate Change (IPCC) concludes that local changes in temperature and rainfall will continue to alter the distribution of disease vectors and the risk of MBVDs [35]. Hence, climate change can increase the elevational range and transmission season especially in cooler areas of the HKH region where the minimum temperature is limiting disease transmission. However, besides elevation, other factors like latitude, and interaction between temperature and latitude should also be considered in explaining the shift of species distribution towards higher elevation [36]. *Aedes aegypti* mosquitoes are also genetically diverse which explains their rapid and highly successful adaptation to human habitats [37]. Furthermore, a recent study has suggested that different mosquito vectors co-occur with the same virus at different temperatures despite significant overlap in vector temperature ranges and they are mostly able to transmit the virus in their optimal climate range [38]. Additionally, the mosquito species adapted to their native environment cause a greater risk and there might be possible under-estimation of threat from mosquito species indigenous to the temperate regions [38]. Therefore, the subsequent spread of mosquitoes at higher elevation is related to both, change in climate as well as the adaptive flexibility of the mosquito.

### Burden of mosquito-borne viral diseases in Asia and the Hindu-Kush Himalayas

Asia has been a hotspot of dengue fever and chikungunya mainly due to its dense human population, unplanned urbanization and poverty. About 70% of 96 million clinically apparent DENV infections in 2010 were contributed by Asia and 34% by India alone [39]. Periodic outbreaks of chikungunya have been reported from Asia since 1960s [40]. Since its arrival in Asia, CHIKV has become widely established and caused major outbreaks like the one in India with more than 1.4 million cases in 2006 [41], and others in countries such as Indonesia, Maldives, Myanmar and Thailand between 2006 and 2007 [42]. The presence of CHIKV in Nepal was confirmed in 2013 [43]. In 2017, a rapid outbreak of CHIKV have also been reported in Bangladesh [44] and Pakistan [45]. Regarding ZIKV, the US Centers for Disease Control and Prevention (CDC) identified 12 countries from Asia as areas with ZIKV risk in September 2016, of which four countries, Bangladesh, Myanmar, India and Pakistan, are in the HKH region [46].

### The urgent need for updated travel advisory about Zika virus transmission

The travel warning issued in March 2016 by the CDC, based on a spatial analysis of the probability of occurrence of *Ae. aegypti*, recommended that pregnant women should avoid travel to elevations below 2000 m in countries with active ZIKV transmission and the risk of ZIKV transmission above 2000 m was considered minimal [47]. However, few cases (e.g., Columbia) of ZIKV have been found in areas above 2000 m [48]. The limited number of cases reported in areas above 2000 m should not discount the threats of ZIKV transmission. The vectorial potential of *Aedes* can be used as a proxy for estimating the risk for ZIKV. *Ae. aegypti vectors* have been reported in areas above 2000 m. For example, across 16 countries in the Americas, 1.1% of reported cases of dengue were in areas above 2000 m [49]. Similarly, in Mexico where *Ae. aegypti* mosquitoes were not reported at greater than 1700 m elevation before, a moderate abundance of these mosquitoes has recently been recorded up to 1700 m and occasionally at elevation between 1700 and 2130 m extending the known elevation range of the vector by more than 300 m [50]. Similar observations have been reported from other countries like Peru, Colombia, and Venezuela [51]. However, the presence of *Aedes* vectors does not necessarily mean the transmission of ZIKV.

Over the last decade, DENV and CHIKV transmissions by *Ae. aegypti* have extended to the Himalayan countries of Bhutan and Nepal [52–55]. ZIKV could follow in the footsteps of these viruses in the HKH region as described elsewhere [56, 57]. In endemic areas, underreporting or misreporting of ZIKV infection is likely in settings which lack adequate diagnostic facilities. In the countries of the HKH region that have MBVD transmission at elevations close to or above 2000 m, however, efforts to understand the risk of ZIKV are completely missing [58–61]. The risk of a ZIKV epidemic can increase along with the vectorial capacity of mosquitoes that is higher during the warmer monsoon season, usually from June to September. Like the dengue and chikungunya epidemics, Zika epidemics are also expected to occur mainly during the post-monsoon season which extends from September to November in the HKH region.

The above discussion drives home a very important message of the need to integrate climate (e.g., temperature, rainfall, humidity) with health data (e.g., morbidity, co-morbidity and mortality estimates related to MBVDs) to generate travel and other health-related advisories that would ultimately benefit the larger HKH community. In this regard, efforts should be made by respective country-level departments to generate relevant information that will be useful towards further investigating the link between impact of climate change on mosquito-vector transmission patterns and human health in the region.

### Transnational, bottom-up “one health” strategies for the Hindu-Kush Himalayas

Knowing that ZIKV, its vectors, and favorable environmental conditions for their spread are present in the HKH region, there should be no place for complacency. Poverty, rapid urbanization, in- and out-migration of people between endemic and non-endemic areas are increasing the number of imported ZIKV cases, and massive climate change may shape the complexity of an intensified and increased risk of ZIKV transmission in the HKH region. In addition, a study conducted in the Central Himalayas revealed low knowledge levels about the day biting behavior of *Ae. aegypti* and *Ae. albopictus* as well as prevention and control measures of DENV infection [62]. Similar results in the region may be expected for other MBVDs including ZIKV. Local communities and tourists travelling to the Himalayas are not yet aware of the risk of MBVDs and control measures such as avoiding mosquito-human contact, destroying breeding sites of mosquitoes and attempting timely diagnosis of any febrile illness, but should become so soon. Additionally, the WHO, the International Center for Integrated Mountain Development (ICIMOD) and research institutes of the region should engage the Ministries of Health and Environment of HKH countries to map the regional risk of ZIKV and prepare for outbreaks. In March 2016, WHO released its response framework and joint operations plan to guide the current international response to the spread of ZIKV and associated conditions. According to the plan [5], the response strategy requires: surveillance of ZIKV and associated complications; involvement of communities to both respond to the virus and effectively communicate about the risks; improvement of vector control; providing care for the affected people; fast tracking further investigation and research about associated complications, developing diagnostics, vaccines and therapeutics; and mobilizing/coordinating partners, experts and resources as required.

To prepare for the possible ZIKV outbreaks, Asia and the HKH region can also learn from the success stories and strategies adopted by other regions and countries in preventing ZIKV and associated complications. Cuba is one of the few countries in the Western hemisphere that anticipated the arrival of the virus to mitigate the impact of ZIKV [63]. Cuba applied prevention efforts coordinated by local health clinics, which deployed mosquito-control workers such that every house in the country would be visited by health professionals to check for mosquitoes, larvae and any standing water that could serve as a breeding ground for mosquitoes [63]. There were systems in place to closely track people who traveled abroad, and screening of pregnant women [63]. Governments in Colombia, Jamaica, El Salvador and Panama released official statement advising women to postpone their pregnancy to avoid possible risk of ZIKV and its complications [64]. In Brazil, military forces have been deployed to educate people on vector control through visiting all houses in the country. Similarly, several other campaigns have been launched by the government to mobilize people to act against mosquitoes [64]. China adopted a rapid Mini8 portable PCR system in airports to test travelers from countries with known outbreaks who have clinical symptoms like fever [65]. The US state, Florida, has set an example through its investment on research on mosquitoes and mosquito-borne diseases and identifying effective, efficient and ecologically sound mosquito control measures (especially for the Culex-borne species) such as arbovirus surveillance program, improved pesticide application, Rotational Impoundment Management (RIM) etc. [66]. Grassroots advocacy and local community participation strategies should be part of an inclusive bottom-up strategy to reduce the breeding sites of mosquitoes. These efforts should be streamlined with the efforts to promote safer sex practices and access to reproductive and sexual health counseling that are already in place to prevent diseases like HIV/AIDS and other sexually transmitted diseases.

“An outbreak anywhere is potentially a threat everywhere” [67]; to ensure international health security, therefore, “all efforts to prevent, detect, and respond to Zika virus” ought to be intensified now in the HKH region. Responding to ZIKV in this region at a transnational level is however far more challenging than one might imagine due to its unique geopolitical scenario, a population of more than 1.5 billion in the foothills and plains of the HKH alone, and a huge number of people migrating across international borders. There is an urgent need of regional collaborative approaches to harmonize surveillance and vector control, and share epidemiological information [68]. Along these lines, we request precedence of health diplomacy over foreign policy in the wake of imminent risk of ZIKV including DENV and CHIKV in the HKH region. Only concerted transnational and transdisciplinary research actions that embrace a “One Health” concept and strengthen regional as well as north-south collaborations**,** hold sufficient promise for improving evidence-based decision-making fast enough to impact the prevention and control of ZIKV and other MBVDs in the fragile and highly vulnerable HKH region.

## Conclusions

The future control strategies for DENV, CHIKV and ZIKV should be considered in tandem with the threat to human well-being that is posed by other emerging and re-emerging vector-borne and zoonotic diseases, and by the continuing urgent need to strengthen public primary healthcare systems in the region. Furthermore, political leadership at regional, national, state and local levels is of paramount importance. In addition to health authorities, the highest government authority at each level should lead and hold all sectors (such as health, water, sanitation, education and social services) accountable on this topic in a coordinated manner. There is also an urgent need in the region for countries to increase their budgetary allocation on healthcare that is critical towards enhancing infrastructure support, testing and reporting systems as well as advancing research to develop pragmatic mitigation strategies required to control the increasing challenges from ZIKV and other vector-borne diseases. Special consideration should be given to cross-border surveillance and coordination mechanisms. Respective ministries from health, tourism and external/foreign affairs from HKH countries should agree to extend all possible support by preventing ZIKV from become an epidemic. Finally, such one health approaches should integrate the social, economic, environmental, cultural, behavioral as well as climatic determinants of health and adequately incorporate human rights, equity and gender equality perspectives.

## Additional file

Additional file 1: Multilingual abstract in the five official working languages of the United Nations. (PDF 337 kb)

## Abbreviations

CHIKV: Chikungunya virus
DENV: Dengue virus
HKH: Hindu Kush Himalayan
JE: Japanese encephalitis
MBVDs: Mosquito-borne viral diseases
ZIKV: Zika virus
