# Supplementary material for: Threats of Zika virus transmission for Asia and its Hindu-Kush Himalayan region
Source: Infect Dis Poverty. 2018 May 15;7:40. doi: 10.1186/s40249-018-0426-3 (PMC5952373; doi:10.1186/s40249-018-0426-3)
Supplement: Supplementary file 1 — Multilingual abstract in the five official working languages of the United Nations. (PDF 337 kb) [file 40249_2018_426_MOESM1_ESM.pdf]

Translation of the abstract into the five official working languages of the United Nations

تواجه آسيا ومنطقة هندوكوش في الهيمالايا التابعة لها أخطار انتقال فايروس زيكا لارجاءها.

قدمه: مغنات دهلال، وسوشما دهيمال، ومانديرا لاميكان دهيمال، وشيفا راج ميشرا، وكيم بركي، وكريشنا كومار أريال، وعبيد الحق، ومحمد إقبال كبير، وبراديب جوين، وعظيم محمود بت، وهارابان هارابان، وتشي يونغ ليو، وكورديا تشو، ودورين مونتاج، ودايفيد الكسندر جرونبيرج، وباسو ديف باندي، وأولريتش كوتش وروث مولر.

الخلاصة

إن آسيا ومنطقة هندوكوش في الهيمالايا التابعة لها (HKH) تحديدًا سريعة التأثر بالتغير البيئي، وخاصةً التغيرات المناخية والتغيرات في مجال استغلال الأراضي والتي تتأثر كذلك بفعل النمو السكاني السريع، وارتفاع معدل الفقر والتنمية غير المستدامة. فقد كانت آسيا مركزًا لتفشي حمى الضنك والشيكونغونيا ويعود ذلك بصفة رئيسية إلى كثافتها السكانية الكبيرة، والتوسع الحضري العشوائي والفقر. ففي دوامة التوسع الحضري تنتقل فيروسات حمى الضنك (DENV) والشيكونغونيا (CHIKV) عبر بعوضة الحمى الصفراءوالبعوضة المنقطة بالأبيضالتي تُعد من البعوض الحامل لفايروس زيكا (ZIKV) كذلك.وخلال العقد الماضي، فإن انتقال فايروسات حمى الضنك (DENV) والشيكونغونيا (CHIKV) عبربعوضة الحمى الصفراءتوسعت لتشمل بلدان في جبال الهيمالايا كالبوتان والنيبال وقد يسير فايروس زيكا (ZIKV) على خطى هذه الفيروسات في منطقة هندوكوش في الهيمالايا (HKH) . إذ أن التوزيع المعتمد للمناطق التي يتواجد بها البعوض الذي يعض الإنسان بعوضة الحمى الصفراءوالبعوض الناقل للفيروسات وضف عليها سذاجة السكان دون مناعة ضد أماكن انتشار فايروس زيكا (ZIKV) فبذلك تكون منطقة هندوكوش في الهيمالايا أكثر عرضة لخطر فايروس زيكا (ZIKV). فبعض البلدان التي تقع بمنطقة هندوكوش في الهيمالايا (HKH) قد أبلغت مُسبقاً بوجود حالات إصابة بفيروس زيكا (ZIKV). فقد قمنا بتوثيق الخطر المتزايد من انتشار فايروس زيكا (ZIKV) في آسيا ومنطقة هندوكوش في الهيمالايا (HKH) التابعة لها بسبب وجود أعداد كبيرة من البعوض الناقل للأمراض والذي يعض الإنسان وانتشاره الواسع، والتغير المناخي، والفقر، وظهور عدد من حالات الإصابة بين السكان الأصليين في المنطقة، والعدد المتزايد لحالات الإصابة، وسذاجة السكان دون مناعة ضد فايروس (ZIKV). فإن تفشي الفايروس في أي مكان يُشكل خطرًا محتملاً في كل مكان. وبالتالي، لضمان الأمن الصحي العالمي، يجدر الآن تعزيز جميع الجهود الرامية إلى منع فايروس زيكا (ZIKV)، واكتشافه، والتصدي له في آسيا وفي منطقة هندوكوش بالهيمالايا (HKH) التابعة لها. وللإستعداد للاحتواء حالات التفشي المحتملة لفايروس زيكا (ZIKV)، يمكن للمناطق بآسيا ومنطقة هندوكوش بالهيمالايا (HKH) أن تتعلم من قصص النجاح والاستراتيجيات التي اتبعتها المناطق والدول الأخرى لمنع فايروس زيكا (ZIKV) والمضاعفات المرتبطة به. إذ ينبغي النظر إلى الاستراتيجيات المستقبلية لمكافحة فايروسات حمى الضنك (DENV)، والشيكونغونيا (CHIKV)، وفايروس زيكا (ZIKV) معاً في آن واحد مع الأخطار التي تهدد رفاه الإنسان والتي تتسبب بها الأمراض الناشئة والمتكررة المنقولة من الحشرات والأمراض الحيوانية المصدر، ومع استمرار الحاجة الماسة لتقوية أنظمة الرعاية الصحية الأولية العامة بالمنطقة.

Translated from English version into Arabic by Bashaier Allam and Heba Kandel, through

TRANSLATORS
WITHOUT BORDERS

寨卡病毒传播对亚洲及其兴都-库什喜马拉雅地区的威胁

摘要

亚洲及其兴都-库什喜马拉雅(HKH)地区人口快速增长、贫困化程度高和不可持续发展导致了气候和土地使用方式发生了变化，进一步恶化该地区应对自然环境变化的能力。由于人口稠密、非规划性城市化和贫穷，部分亚洲地区已成为登革热和基孔肯雅热的流行区。在城市

循环系统中，登革热病毒(DENV)和基孔肯雅病毒(CHIKV) 经由埃及伊蚊和白纹伊蚊传播，同时，它们也是寨卡病毒(ZIKV)的有效载体。在过去的十年中，经由埃及伊蚊传播的 DENV 和 CHIKV 已经扩散到不丹和尼泊尔，而 ZIKV 也可会随这些病毒传播到兴都-库什喜马拉雅地区。在这儿，叮人伊蚊媒介的分布已趋于稳定，但当地人对 ZIKV 免疫力较低，因此感染 ZIKV 的风险较高。兴都-库什喜马拉雅地区的一些国家已经报道过 ZIKV 病例。叮人蚊媒的高丰度和宽分布、气候的变化、贫穷的现状、本地病例的出现、输入性病例数量的增加以及寨卡病毒免疫力缺失的本地人群，加剧了 ZIKV 在亚洲及其兴都-库什喜马拉雅地区的传播风险。任何一处疫情的暴发都会对其他地区造成潜在的威胁。因此，为确保国际卫生安全，亚洲及其兴都-库什喜马拉雅地区应加强对 ZIKV 的预防、检测和响应。为预防 ZIKV 疫情暴发，亚洲及其兴都库什-喜马拉雅地区还可借鉴其他地区和国家在预防寨卡病毒及其相关并发症方面的成功案例和策略。DENV、CHIKV 和 ZIKV 的下一步防控策略，应该同步纳入威胁人类福祉的其他新发、复现的媒介疾病和人畜共患病的防控需求，同时迫切需要强化当地的公共基础医疗卫生系统。

Translated from English version into Chinese by Fan Yang, edited by Jin Chen

CHINESE CENTER FOR DISEASE CONTROL AND PREVENTION
NATIONAL INSTITUTE OF PARASITIC DISEASES

Menaces de transmission du virus Zika en Asie et dans l'aire Hindou Kouch-Himalaya

Meghnath Dhimal, Sushma Dahal, Mandira Lamichhane Dhimal, Shiva Raj Mishra, Khem B Karki, Krishna Kumar Aryal, Ubydul Haque, Md Iqbal Kabir, Pradeep Guin, Azeem Mehmood Butt, Harapan Harapan, Qi-Yong Liu, Cordia Chu, Doreen Montag, David Alexander Groneberg, Basu Dev Pandey, Ulrich Kuch et Ruth Müller

Résumé

L’Asie, et notamment l'aire Hindou Kouch-Himalaya (HKH), est particulièrement vulnérable aux changements environnementaux, en particulier ceux du climat et de l’usage des terres, doublés d’une croissance démographique rapide, d’un niveau élevé de pauvreté et d’un développement non durable. L'Asie est un important foyer de dengue et de chikungunya, à cause notamment de sa forte densité de population, de son urbanisation anarchique et de sa pauvreté. Dans leur cycle urbain, le virus de la dengue (DENV) et celui du chikungunya (CHIKV) sont transmis par les moustiques *Aedes aegypti* et *Ae. albopictus* , qui sont également des vecteurs compétents du virus Zika (ZIKV). Ces dix dernières années, la transmission des virus de la dengue et du chikungunya par *Ae. aegypti* a progressé jusqu’aux pays himalayens du Bhoutan et du Népal ; le virus Zika pourrait suivre dans leur sillage, jusqu’à l'aire Hindou Kouch-Himalaya. La présence déjà établie de moustiques vecteurs du genre *Aedes* piquant les humains et une population naïve, dépourvue d’immunité contre le virus Zika, créent un risque important d’infection dans cette région, où certains pays ont déjà rapporté des cas de maladie dus à ce virus. Nous avons relevé les signes d’une menace croissante du virus Zika en Asie et dans l'aire HKH à cause de l’abondance et de la large distribution de moustiques vecteurs piquant les humains, du changement climatique, de la pauvreté, de cas indigènes dans la région, du nombre croissant de cas importés et de la population naïve, dépourvue d'immunité contre le virus. L’apparition d’un foyer de contagion à un endroit peut créer une menace partout dans le monde.

Afin d’assurer la sécurité internationale, les mesures de prévention, de détection et de lutte contre le virus Zika doivent donc être intensifiées dès à présent en Asie et dans l'aire Hindou Kouch-Himalaya. Afin de se préparer à des épidémies possibles de virus Zika, l’Asie et l'aire HKH peuvent aussi tirer les enseignements des stratégies adoptées avec succès par d’autres régions et pays pour prévenir les infections par ce virus et leurs complications. Les stratégies futures de lutte contre les virus de la dengue, du chikungunya et Zika doivent être menées de front avec la menace sanitaire posée par d’autres maladies vectorielles et zoonotiques émergentes et récurrentes et avec le renforcement des systèmes de soins de santé primaires dans la région, qui s’impose d’urgence.

Translated from English version into French by Suzanne Assenat and Elisa Griffin, through

TRANSLATORS
WITHOUT BORDERS

Опасность распространения вируса Зика для Азии и ее гималайского региона Гиндукуш

Медхнат Дхимал, Сушма Дахал, Мандира Ламичхане Дхимал, Шива Радж Мишра, Кхем Б. Карки, Кришна Кумар Ариал, Юбидул Хаке, Мд Икбал Кабир, Прадип Гуин, Азеем Мехмуд Бутт, Харапан Харапан, Ци-Юн Лю, Кордиа Чу, Дорин Монтаг, Давид Александр Гроунберг, Басу Дев Пандей, Ульрих Кух и Рут Мюллер

Реферат

Азия и ее гималайский регион Гиндукуш (ГКГ) особенно уязвимы к изменениям окружающей среды, в частности изменениям климата и землепользования, на которые, в свою очередь, влияют быстрый рост населения, высокий уровень бедности и истощительная разработка ресурсов. В Азии широко распространены лихорадка денге и чикунгунья, преимущественно из-за высокой плотности населения, стихийной урбанизации и бедности. В городской среде вирус денге (ДЕНВ) и вирус чикунгунья (ЧИКВ) передаются *комаром жёлтолихорадочным* (*Aedes aegypti*) и *кусакой бело-пестрым* (*Ae. albopictus*), которые также являются переносчиками вируса Зика (ЗИКВ). В последнее десятилетие распространение вирусов ДЕНВ и ЧИКВ посредством *Ae. aegypti* достигло гималайских стран Бутана и Непала, открыв тем самым возможность для вируса ЗИКВ проникнуть в регион ГКГ. Уже установлено, что распространение комаров-переносчиков инфекции *Aedes* и отсутствие у ранее не получавшего лечения населения иммунитета против вируса ЗИКВ, повышает уровень риска заражения вирусом ЗИКВ в регионе ГКГ. Некоторые страны региона ГКГ уже сообщили о случаях заражения вирусом ЗИКВ. Мы зафиксировали растущую угрозу вируса ЗИКВ в Азии и регионе ГКГ из-за многочисленности и широкого распространения комаров-переносчиков инфекции, изменения климата, бедности, положения коренных народов в регионе, увеличения числа импортируемых случаев заражения и отсутствия у местного населения иммунитета против ЗИКВ. Вспышка заболевания в любом районе является потенциальной угрозой для всего мира. Следовательно, для обеспечения международной

безопасности здоровья в Азии и регионе ГКГ необходимо усилить все меры по предотвращению, обнаружению и лечению вируса ЗИКВ. Для подготовки к возможным вспышкам вируса ЗИКВ Азия и регион ГКГ могут руководствоваться стратегиями и успешным опытом других регионов и стран по борьбе с вирусом ЗИКВ и связанными с ним осложнениями. Будущие стратегии контроля над вирусами денге, чикунгуньи и Зика должны применяться совместно с борьбой с новыми трансмиссионными и зоонозными заболеваниями или заболеваниями, которые ранее находились под контролем, а также продолжением улучшения систем первичной медико-санитарной помощи в регионе.

Translated from English version into Russian by Oksana Rozhko and Ekaterina_Rugg, through

TRANSLATORS
WITHOUT BORDERS

Riesgo de transmisión del virus Zika en Asia y en la región del Hindu Kush-Himalaya

Meghnath Dhimal, Sushma Dahal, Mandira Lamichhane Dhimal, Shiva Raj Mishra, Khem B Karki, Krishna Kumar Aryal, Ubydul Haque, Md Iqbal Kabir, Pradeep Guin, Azeem Mehmood Butt, Harapan Harapan, Qi-Yong Liu, Cordia Chu, Doreen Montag, David Alexander Groneberg, Basu Dev Pandey, Ulrich Kuch and Ruth Müller

Resumen

Asia y su región del Hindu Kush-Himalaya (HKM) son especialmente vulnerables al cambio ambiental, en concreto a los cambios en el clima y en el uso de tierras influidos por el rápido crecimiento de la población, el elevado nivel de pobreza y el desarrollo no sostenible. Asia ha sido un punto caliente para la fiebre del dengue y el chikunguña, sobre todo debido a la densa población, al desarrollo urbanístico no planeado y a la pobreza. En un ciclo urbano, el virus del dengue (DENV) y del chikunguña (CHIKV) se transmiten a través de los mosquitos *Aedes aegypti* y*Ae. albopictus* que también son vectores capaces de transmitir el virus Zika (ZIKV). Durante la última década, las transmisiones del DENV y del CHIKV mediante *Ae. aegypti* se han extendido a los países del Himalaya, Bhutan y Nepal, y el ZIKV ha podido seguir los pasos de estos virus hastala región del HKH. La distribución establecida del mosquito *Aedes* , el cual pica a seres humanos y es vector del virus, junto con una población ingenua y sin inmunidad contra el ZIKV ponen a la región del HKH en un elevado riesgo de contraer ZIKV. Algunos de los países de la región HKH ya han confirmado casos de ZIKV. Hemos confirmado un aumento del riesgo de ZIKV en Asia y en su región del HKH debido a la alta densidad y a la amplia distribución de los mosquitos vectores que pican a seres humanos, al cambio climático, a la pobreza, a la incidencia de casos autóctonos en la región, al incremento en el número de casos importados y a una ingenua población sin inmunidad contra el ZIKV. Un brote en cualquier lugar es potencialmente una amenaza en todas partes. Por lo tanto, con el fin de asegurar la seguridad de la salud internacional, en estos momentos todas las campañas para prevenir, detectar y responder al ZIKV se deben intensificar en Asia y en su región del HKH. Para estar listos frente a posibles brotes de ZIKV, Asia y la región del HKH pueden, a su vez, tomar nota

de los casos exitosos y de las estrategias adoptadas en otras regiones y en otros países para prevenir el ZIKV y las complicaciones asociadas. Las medidas de control que se tomen en un futuro frente al DENV, CHIKV y ZIKV se deben considerar de forma conjunta con la amenaza al bienestar de los seres humanos que puedan suponer otras enfermedades transmitidas por vectores y enfermedades zoonóticas, emergentes o recurrentes, y con la necesidad urgente de reforzar los sistemas de salud pública primaria en la región.

Translated from English version into Spanish by Patricia Blazquez and Ana Usán, through

TRANSLATORS
WITHOUT BORDERS
